# PdtaS Deficiency Affects Resistance of Mycobacteria to Ribosome Targeting Antibiotics

**DOI:** 10.3389/fmicb.2017.02145

**Published:** 2017-11-03

**Authors:** Karolina Dadura, Renata Płocińska, Anna Rumijowska-Galewicz, Przemysław Płociński, Anna Żaczek, Bożena Dziadek, Andrzej Zaborowski, Jarosław Dziadek

**Affiliations:** ^1^Institute for Medical Biology, Polish Academy of Sciences, Łódź, Poland; ^2^Department of Biochemistry and Cell Biology, University of Rzeszów, Rzeszów, Poland; ^3^Department of Immunoparasitology, University of Łódź, Łódź, Poland

**Keywords:** signal transduction, ribosome, antibiotic resistance, streptomycin, aminoglycosides, tuberculosis

## Abstract

Two-component regulatory systems (TCSSs) are key regulatory elements responsible for the adaptation of bacteria to environmental stresses. A classical TCSS is typically comprised of a sensory histidine kinase and a corresponding response regulator. Here, we used homologous recombination to construct a *Mycobacterium smegmatis* mutant defective in the synthesis of cytosolic histidine kinase PdtaS (Msmeg_1918). The resulting Δ*pdtaS* mutant strain was tested in the Phenotype Microarray screening system, which allowed us to identify aminoglycoside antibiotic sensitivity, tetracyclines antibiotic resistance as well as membrane transport and respiration, as the main processes affected by removal of *pdtaS*. The antibiotic sensitivity profiles were confirmed by survival assessment and complementation studies. To gain insight into the molecular mechanisms responsible for the observed phenotype, we compared ribosomal RNA and protein profiles of the mutant and wild-type strains. We carried out Northern blotting and qRT-PCR to compare rRNA levels and analyzed ribosome sedimentation patterns of the wild-type and mutant strains on sucrose gradients. Isolated ribosomes were further used to estimate relative abundance of individual proteins in the ribosomal subunits using label free mass spectrometry analysis. Additionally, the Δ*pdtaS* mutant revealed lower activity of the respiratory chain as measured by the rate of TTC (triphenyltetrazolium chloride) reduction, while at the same time showing only insignificant changes in the uptake of aminoglycosides. We postulate that deficiency of PdtaS affects the oxidative respiration rates and ribosomal composition causing relevant changes to intrinsic resistance or susceptibility to antibiotics targeting ribosomes, which are commonly used to treat mycobacterial infections.

## Introduction

The genus *Mycobacterium* possesses pathogens known to cause serious diseases in mammals, including tuberculosis (*Mycobacterium tuberculosis*) and leprosy (*Mycobacterium leprae*) in humans as well as saprophytes or non-pathogenic soil bacteria, e.g., *Mycobacterium smegmatis*. *M. smegmatis* is a commonly used model species of Mycobacteria that is easy to work with, with a fast doubling time and only requires a biosafety level 1 laboratory. This species shares more than 2000 homologous genes with *M. tuberculosis* and shares the same peculiar cell wall structure of *M. tuberculosis* and other mycobacterial species (King, 2003). It is also capable of oxidizing carbon monoxide aerobically, as is *M. tuberculosis*.

The evolutionary success of both fast- and slow-growing mycobacteria is based on their ability to adapt to changing growth environments. The bacterium utilizes two-component regulatory systems (TCSSs) to sense and adequately respond to the changing growth conditions. Each system is typically comprised of a sensory histidine kinase that is capable of autophosphorylation when receiving an appropriate stimulus from the environment and subsequently transfers the phosphor group onto its partner protein—the response regulator. The regulatory protein is typically a transcription factor that binds within the promoter regions of a defined set of genes, promoting or repressing their expression. *M. tuberculosis* possesses 11 pairs of genetically linked TCSSs, as well as five uncoupled orphan regulators and at least two orphan histidine kinases (Bretl et al., 2011). Comparative genomic analyses of the 11 genetically linked TCSSs in *M. tuberculosis* indicate that homologs of these genes exist also in *M. smegmatis* (Bretl et al., 2011). Various TCSSs are already implicated in altering the natural antibiotic resistance patterns in mycobacteria (Zhou et al., 2012), while MtrAB and PrrAB systems have been found to be essential for *M. tuberculosis* survival (Rajagopalan et al., 2010; Haydel et al., 2012). Most response regulators, like MtrA, act as transcriptional regulators; however, in some rare instances, the response regulator can specialize to fulfill other tasks in the cell. This is the case for *Escherichia coli*’s CheY, which, upon phosphorylation by its partner sensory kinase, CheA, acts as a flagellar motor switch, binding to the flagella, activating its movement and allowing bacteria to travel to more favorable, nutrient rich niches (Dyer et al., 2009). Another extraordinary group of response regulators includes the ANTAR (AmiR and NasR Transcriptional Antitermination Regulator) domain containing proteins. These regulators are believed to form specific antiterminator structures with the newly transcribed RNA, binding stem loop structures or sequences present either in the 5′ UTR regions of regulated transcripts or within the polycistronic RNA species, preventing premature RNA polymerase disengagement (Shu and Zhulin, 2002; Boudes et al., 2012). One of these putative antiterminator proteins, PdtaR (Rv1626), exists in *M. tuberculosis* and is known to be phosphorylated by its cognate sensory kinase PdtaS (Rv3220c) (Morth et al., 2005). The role of PdtaS-mediated phosphorylation remains unclear, as many PdtaR-like antiterminators lack the response regulator domain in other bacterial species and act regardless of their phosphorylation status (Ramesh et al., 2012). Here, the directed KO mutant of *M. smegmatis*Δ*pdtaS* (*msmeg_1918*) was engineered to investigate the role of PdtaS/R TCSS in resistance to antimicrobial compounds. The Phenotypic Microarray system (BIOLOG) was used to evaluate the sensitivity of the Δ*pdtaS* mutant to 240 different chemical agents including antibiotics, detergents and toxic ions. A strong phenotype was identified for aminoglycoside antibiotics, as well as membrane transport and respiration inhibitors. The molecular mechanism of PdtaS-dependent sensitization of mycobacteria to aminoglycosides is discussed in this paper.

## Materials and Methods

### Bacterial Strains and Growth Conditions

The *M. smegmatis* mc^2^155 (Snapper et al., 1990) strains used in this study were cultured in Middlebrook 7H10 agar or 7H9 broth supplemented with 10% OADC enrichment (oleic acid albumin dextrose catalase), 0.05% Tween 80 (pH 6.0–6.2), and antibiotics: 50 μg/ml hygromycin (Hyg), or 25 μg/ml kanamycin (Kan).

### Gene Cloning Strategies

Standard molecular biology protocols were used for all cloning procedures (Sambrook and Russell, 2001). All PCR products were obtained using thermostable AccuPrime*Pfx* DNA polymerase (Invitrogen), cloned initially into a blunt vector (pJET 1.2/blunt; Thermo Fisher Scientific), sequenced and then released by digestion with appropriate restriction enzymes before cloning into the final vectors. To facilitate subcloning into expression vectors, restriction enzyme recognition sites were incorporated into the sequence of the primers. The plasmids used in this work are listed in Supplementary Table S1.

### Construction of Gene Replacement Vectors and Complementation Plasmids

A suicidal recombination delivery vector based on p2NIL was used to generate unmarked deletions of *pdtaS* gene in *M. smegmatis* (Parish and Stoker, 2000). The recombination vectors carried the 5′*pdtaS* upstream region of 1136 bp and the first 79 bp of the *pdtaS* gene tagged to the 3′ part of the *pdtaS* gene (807 bp), followed by 448 bp of the *pdtaS* downstream regions—pKD4. PCR products carrying 5′ and 3′ fragments of the gene were ligated out of frame, such that the resulting Δ*pdtaS* gene encoded a short, non-functional protein. Finally, the PacI screening cassette from pGOAL17 was inserted into the constructs, resulting in the suicide delivery vector pKD5 which was used to engineer the directed *M. smegmatis* mutant as described previously (Dziadek et al., 2002; Brzostek et al., 2009; Plocinska et al., 2012). The required plasmids and primers are listed in Supplementary Table S1.

To prepare the complementation plasmid, intact *pdtaS* gene from *M. smegmatis* was PCR amplified (using primers listed in Supplementary Table S1) and cloned into BamHI–HindIII sites of pKW08Lx vector downstream of the *P_tet_* promoter (Supplementary Table S1). Next, the gene and the *P_tet_* promoter were excised from the resulting vector (pKL5) with HindIII and XbaI and cloned into the integrating vector pKW08Lx-Int, generating pKD10.

### Disruption of *M. smegmatis pdtaS*

The protocol of Parish and Stoker (2000) was used to disrupt the *M. smegmatis pdtaS* gene at its native loci on the chromosome. The suicidal recombination plasmid DNA (pKD5) was treated with NaOH (0.2 mM) and integrated into the *M. smegmatis* chromosome by homologous recombination. The resulting single crossover recombinant (SCO) mutant colonies were blue, Kan^R^ and sensitive to sucrose. The site of recombination was confirmed by PCR and Southern hybridization (data not shown). The SCO strains were further processed to select for double crossover (DCO) mutants that were white, Kan^S^ and resistant to sucrose (2%). The genotypes of obtained mutant DCO strains were confirmed by PCR and Southern hybridization. Probes to hybridize to the *pdtaS* gene were generated by PCR by labeling with a non-radioactive primer extension system (DIG-labeling system, Amersham). Primers used for PCR amplification are listed in Supplementary Table S1.

### Phenotypic Microarrays

The *M. smegmatis* wild-type strain and Δ*pdtaS* mutant were initially grown on 7H10/OADC at 37°C. Colonies were suspended in inoculation fluid (IF0a - BIOLOG protocol) using a sterile swab to a final transmittance of 81%. The redox indicator tetrazolium violet was added to every cell suspension according to the PM BIOLOG protocol appropriate for PM plates. Next, every well of the PM11-PM20 plates was inoculated by adding 100 μl of the suspension of bacterial cells. The PM11-PM20 plates contained 240 different chemicals, each at four different concentrations. Inoculated PM plates were incubated at 37°C in a BIOLOG Omnilog incubator, which allowed for collection of a set of growth kinetic related measurements every 15 min during the 72 h of incubation time. The bacterial growth was estimated as a rate of reduction of the tetrazolium violet to a purple colored formazan proportionally to the respiration of bacteria. The intensity of the purple color was recorded as a value and plotted by the software in an automated manner. Then, the growth of the wild-type and mutant strains in the presence of a given chemical was compared by overlapping the area under the curve (AUC) for respective conditions. AUC values from each well for wild-type and mutant strains collected in at least two independent experiments were exported from the BIOLOG software into an Excel file format and analyzed. The difference in AUC between the wild-type and mutant strains was due to different growth dynamics or different survival rates in the presence of tested antibiotics, detergents and toxic ions.

### Spot Dilution Assay

Exponentially grown *M. smegmatis* cells were initially diluted to an optical density OD_600_ of 0.5- and 10-fold serial dilutions were prepared. 10 μl of cells from each dilution (10^0,^10^-1^, 10^-2^, 10^-3^, 10^-4^) were spotted onto 7H10 solid medium containing OADC and different ranges of concentration of antibiotics: apramycin (0.1 – 0.7 μg/ml), sisomicin (0.5 – 2.0 μg/ml), streptomycin (SM-0.3 – 2.0 μg/ml), dihydrostreptomycin (DIH-SM – 0.2 – 1.0 μg/ml), kanamycin (1.0 – 2.5 μg/ml), tetracycline (TET- 0.1 – 1.0 μg/ml). Plates were incubated at 37°C for 3–5 days and photographed. Three independent repetitions were performed.

### Survival Assessment in the Presence of Growth Inhibitors

The survival of *M. smegmatis* Δ*pdtaS* mutant cells grown in liquid medium with the addition of chosen antibiotics was studied using standard CFU methodology. Actively growing cells from the logarithmic stage of growth were diluted in 7H9 medium supplemented with OADC and Tween-80 to an OD_600_ of 0.1. Chosen antibiotics were used at the following final concentrations: apramycin 0.55 μg/ml, sisomicin 2.3 μg/ml, streptomycin 0.5 μg/ml, dihydrostreptomycin 0.5 μg/ml, kanamycin 2.0 μg/ml and tetracycline 0.1 μg/ml, which was based on the results of the spot dilution assay. The kinetics of growth was measured by recording OD_600_ at 3, 6, 9, and 24 h after addition of antibiotics. At the 9 and 24 h time points, 10-fold serial dilutions of cells were spread on 7H10 agar containing OADC. Plates were incubated at 37°C for 3–5 days, colonies were counted and data were plotted using Excel. A Student’s *t*-test was performed to determine the statistical significance between the test and control values. All statistical calculations were performed with help of the SigmaPlot 12.0 software.

### Minimum Inhibitory Concentration (MIC) of Aminoglycoside Antibiotics

In order to determine the MIC values for tested here antibiotics, the microplate Alamar blue assay (MABA test) was employed as described previously (Franzblau et al., 1998), with minor modifications. All microplate Alamar blue assays were performed using 96-well flat-bottom plates (Techno Plastic Products). The standardization of the bacterial cell number used for MABA test is critical for obtaining accurate and reproducible results. Thus, the recommended final inoculum was prepared at 5 × 10^5^ CFU/ml by diluting logarithmic phase grown cultures with 7H9 broth supplemented with 10% OADC and Tween-80 initially to a 0.5 McFarland turbidity and 100X further for inoculation (Luna-Herrera et al., 2003; Wiegand et al., 2008).

The microdilution of antibiotics (kanamycin, streptomycin, dihydrostreptomycin, apramycin, sisomicin, tetracycline) was performed in 96-well plates. Two-fold dilutions of each antibiotic were prepared in the test wells in complete 7H9 broth and the final antibiotic concentration ranges were as follows: kanamycin 6–0.046 μg/ml, streptomycin 5–0.04 μg/ml, dihydrostreptomycin 5–0.04 μg/ml, apramycin 5–0.04 μg/ml and sisomicin 4–0.03 μg/ml, tetracycline 4-0.03 μg/ml. One hundred microliters of each bacterial suspension was added to 100 μl of drug-containing culture medium. The plates were incubated for 48 h at 37°C and this time point 25 μl of Alamar blue solution (Invitrogen) was added to each well. Plates were incubated at 37°C for additional 24 h (Chung et al., 1995; Banfi et al., 2003). After incubation time plates were read for color change from blue to pink and absorbance was measured at 570/600 nm (Benchmark Plus Microplate Spectrophotometer, Bio Rad). Wells containing only bacteria, medium, or antibiotic were used as controls in each plate. MIC was defined as the lowest concentration of antibiotic that prevented color change from blue to pink. The MIC value for each antibiotic was determined from at least three independent experiments.

### The Uptake of Streptomycin

The bacterial cells were cultured in 7H9 medium supplemented with OADC and Tween-80 up to OD_600_ 0.5. Based on the results from the spot dilution assay, 20 ml of mycobacterial cells were exposed to streptomycin at a final concentration of 0.7 μg/ml for 60 and 180 min. After pre-incubation with the tested antibiotic, the bacilli were centrifuged, washed with 7H9 medium and bead-beated twice for 45 s, 6.0 m/s using the Ms disruptor system with Quick prep adapter (MP Biomedicals). The streptomycin uptake by the cells of the wild-type and mutant strains of *M. smegmatis* was monitored with application of the commercially available competitive immunoenzymatic Green Spring Streptomycin ELISA test Kit (Antibodies-online Inc., United States) according to manufacturer’s indications. The mathematical calculations of the SM concentrations in the experimental and control tubercle bacilli cell lysates were based on the standard curve that was established following the procedure recommended by the manufacturer.

### Microplate Alamar Blue Assay

The microplate Alamar blue assay was applied to study respiratory activity of *M. smegmatis* by monitoring resazurin reduction. 200 μl/well (1 × 10^6^ cells/ml) of Δ*pdtaS* and wild-type *M. smegmatis* cells were seeded in triplicate into a 96-well plate. Then, 25 μl of Alamar blue reagent was added into each well directly after inoculation and the plate was incubated at 37°C in a humidified atmosphere. The optical density of the plate was measured at 570 nm and 600 nm at 0, 7, and 11 h of growth using Benchmark Plus Microplate Spectrophotometer (BioRad). Data were plotted using Excel.

### Respiration Activity Assay Using 2,3,5-Triphenyltetrazolium Chloride (TTC)

TTC powder was dissolved in sterile distilled water at a concentration of 5 mg/ml at room temperature, then filtered through 0.22 μm syringe filters. Δ*pdtaS* and wild-type *M. smegmatis* cells from the logarithmic phase of growth were diluted to 1 × 10^6^ cells/ml and seeded into a 96-well plate. Next, TTC at a final concentration of 0.625 mg/ml was added to 200 μl of diluted culture. The plate was incubated at 37°C and red formazan formation was measured at 480 nm at 0, 2, and 7 h timepoints of incubation by Benchmark Plus Microplate Spectrophotometer (BioRad). To determine any significant differences between studied strains the Student’s *t*-test was applied. The statistically significant difference was calculated as (*P* ≤ 0.001).

### RNA Isolation and Northern Blotting Analysis

Total RNA was extracted from the wild-type *M. smegmatis* strain and corresponding Δ*pdtaS* mutant strain as described previously (Pawelczyk et al., 2011). Briefly, 15 ml of cells from logarithmic and stationary phases of growth were centrifuged at 4500 rpm for 10 min at 4°C. The cell pellet was resuspended in 300 μl of DEPC water and 900 μl of Trizol LS reagent (Invitrogen) was added and transferred into screw cap tubes containing 500 μl of 0.1 mm silica spheres (MP Biomedicals). Cells were disrupted twice for 45 s, 6.0 m/s with 5 min intervals on ice using an MP disruptor system with Quick prep adapter (MP Biomedicals). Then, DNase I (Invitrogen) was used to remove DNA contamination according to the manufacturer’s instructions.

The levels of precursor ribosomal RNA species and their processing intermediates were monitored by Northern blotting analysis. Samples of 2 μg of total RNA from two independent biological replicates were resolved in a denaturing 1.2% agarose gel containing formaldehyde in 1X NBC buffer pH 7.5 (50 mM boric acid, 1 mM sodium acetate, 5 mM NaOH) and 0.9% formaldehyde (Szczesny et al., 2010). Gel was transferred onto a Hybond+ nylon membrane by capillary transfer in 20xSSC pH 7.0 (3 M NaCl, 0.3 M sodium citrate) buffer overnight. Next, RNA was immobilized by cross-linking to the membrane with UV260, stained with methylene blue to confirm transfer efficiency and used for hybridization with γ-^32^P-ATP labeled, 20–25 bp long oligonucleotide probes complementary to the transcript tested (for primer sequences see Supplementary Table S1). After extensive washing, the membrane was incubated in a phosphorimaging screen and cassette and the screen was scanned in the Typhoon apparatus to visualize the results.

### Quantitative Real Time (qRT) PCR

The same RNA samples tested for Northern blotting were used for quantitative real time PCR (qRT-PCR) analysis of transcript levels for 16S, 23S and 5S genes. The reverse transcription reaction and qRT-PCR analysis were performed as described previously by Pawelczyk et al. (2011). Briefly, 1 μg of total RNA was reverse transcribed using specific primers to each studied gene (Supplementary Table S1) and the SuperScript III First-Strand Synthesis SuperMix (Invitrogen). qRT-PCR analysis of expression levels of 16S, 23S and 5S genes was performed using the Maxima SYBR green qPCR master mix (Thermo Fisher Scientific). Each reaction mixture at a final volume of 25 μl contained 1 x Maxima SYBR green qPCR master mix, 50 ng of cDNA, and 0.3 M of each primer (see Supplementary Table S1 for primer sequences). The qRT-PCR reaction was carried out in a 7900HT real time PCR system (Applied Biosystems). The three step cycling protocol was used for transcript analysis of 16S, 23S and 5S: initial denaturation at 95°C for 10 min, followed by 40 cycles at 95°C for 15 s (denaturation), 56°C for 30 s (annealing), 72°C for 30 s (extension). Data were collected and acquired during the extension step. The melting curve analysis was performed at the end of each PCR in order to verify the specificity of the generated PCR product. All the reactions were performed in triplicate and three different RNA samples from independent experiments were tested. The results were normalized to 16S, 23S and 5S copy number of the DNA standard curve as described (Gagala et al., 2014). To prepare the calibration standards, the *M. smegmatis* 16S and 23S-5S transcript sequences were cloned into pJET1.2/blunt vector (Thermo Fisher Scientific). The obtained plasmids pKD11 and pKD12 were linearized by restriction digestion with NcoI and ScaI, respectively, and the gene copy number per μl was determined. Then, serial 10-fold dilutions of 2 × 10^10^ copies of each gene were used to make the standard curve.

### Ribosome Analysis

Appropriate mycobacterial strains were grown exponentially in 250 ml of 7H9 media and were pelleted and frozen over a liquid nitrogen bath. When needed, cells where thawed and lysed in lysis buffer containing 20 mM HEPES pH 7.5, 200 mM KCl, 5 mM MgCl_2_, 1% Triton X-100, 2 mM DTT, 5 U/ml of DNase turbo (Thermo Fisher Scientific) using the same procedure as described for total RNA isolation. Cell lysate was precleared by centrifugation at 14,000 rpm for 20 min, passed through a 0.22 μm Ultra free-MC microcentrifuge filter (Millipore) and overlaid onto a freshly prepared continuous 7 – 47% sucrose gradient (Esposito et al., 2010). The samples were spun at 39,000 rpm for 2 h with “no brake” deceleration in a swinging bucket TH641 rotor using Thermo Sorvall WX90 ultracentrifuge and gradients were collected using an AKTA Purifier (GE Healthcare) equipped with UV monitor. Ribosomal spectra were recorded and plotted for comparison.

For comparative proteomic analysis, fractions containing ribosomal subunits separated by their sedimentation indexes were split in half and precipitated using PRM reagent (acidified pyrogallol red). Protein precipitates were either run on an SDS-PAGE gel or directly analyzed using mass spectrometry (MS/MS). The protein gels were inspected visually and the single band that differed the most between the wild-type and mutant isolations was excised and subjected to MS/MS identification. The MS analysis was performed similarly as described elsewhere (Plocinski et al., 2014). Briefly, protein samples were reduced with DTT, alkylated using iodoacetamide and subjected to standard Trypsin digestion. Resulting peptide mixtures were loaded onto RP-18 pre-columns and transferred to a nano-HPLC RP-18 column in acetonitrile gradient. The HPLC system’s outlet was directly connected to the Orbitrap Velos (Thermo Fisher Scientific). The data-dependent mode of data collection was used, which allowed us to switch between Orbitrap MS and LTQ–MS/MS acquisition. Obtained “-.raw” files were next processed by MaxQuant software (v1.3.0.5) using default search parameters against the randomized *M. smegmatis* database and results were exported to Excel format.

### β-Galactosidase Activity Assay

*Mycobacterium smegmatis* Δ*pdtaS* and wild-type cells carrying an integration plasmid expressing *lacZ* gene under the acetamide promoter were grown in broth (7H9, Tween-80, OADC) with addition of SM – 0.7, 0.2, and 0.1 μg/ml, DIH-SM – 0.5, 0.05, and 0.01 μg/ml and TET – 0.1 μg/ml from OD_600_ 0.15 for 6 h to mid-logarithmic phase of growth. The cells from each culture (1 ml) were disrupted using an MP disruptor system with Quick prep adapter (MP Biomedicals), 2 × 45 s, 6.0 m/s with 5 min intervals on ice. Cells were harvested (5 min, 14000 rpm at room temperature) and supernatants were used to determine the β-galactosidase activity according to the protocol by Karimova et al., 2000. Briefly supernatants were mixed with PM2 buffer (70 mM Na_2_HPO_4_X12H_2_0, 30 mM NaH_2_PO_4_XH_2_0, 1 mM MgSO_4_, 0.2 mM MnSO_4_, 100 mM β-mercaptoethanol), reaction was started by addition of 0.4% *o*-nitrophenol-β-galactoside (ONPG) and incubated at 28°C for 20 min. The reaction was terminated by adding sodium carbonate to 0.5 M final concentration. Next, the absorbance at 420 nm was recorded and the enzymatic activity was calculated in relation to the starting OD_600_ of each tested culture.

## Results

### The *pdtaS* Gene Product Is Not Essential for Growth and Survival of *M. smegmatis* Cells

Compared to the genetically linked two component signal transduction systems from mycobacteria, very little is known about the orphaned elements of the signaling cascade in these bacteria. *PdtaS* (*rv3220c*) was previously shown to be a non-essential gene in *M. tuberculosis* and has been disrupted by Parish and collaborators (Parish et al., 2003). To evaluate the role of the PdtaS sensor kinase in mycobacteria, we constructed a *M. smegmatis pdtaS* deletion strain using two-step homologous recombination (Parish and Stoker, 2000). The list of plasmid constructs and primers used in this study is provided in Supplementary Table S1. The obtained single crossover (SCO) recombinants carrying both the wild-type *pdtaS* copy and the Δ*pdtaS* copy with an internal deletion, verified by PCR (data not shown), were screened following the protocol for double crossover selection (DCOs). Our numerous attempts to construct a knockout mutant strain of Δ*pdtaS* in *M. smegmatis* cells were unsuccessful, thus we first generated the conditional *pdtaS* mutant in the SCO background with an *att*B-integrated pKW08 vector carrying the intact *pdtaS* gene under tetracycline promoter (pKD10). The resulting strain was subjected to a second crossover event screening to generate double crossovers and the pKD10 plasmid was later swapped efficiently with an empty pMV306 plasmid, which was finally lost by culturing bacteria without antibiotic pressure. The genotype of Δ*pdtaS M. smegmatis* mutant strain was confirmed by PCR and Southern blot hybridization (Supplementary Figure S1). Analysis of growth kinetics by measurements of optical density of liquid cultures at 600 nm and viability (CFU/ml) for Δ*pdtaS M. smegmatis* cells revealed only modest differences comparing to the wild-type strain when grown in liquid media (Supplementary Figure S2).

### *M. smegmatis* Δ*pdtaS* Mutant Reveals Altered Sensitivity to Aminoglycosides, As Well As to Inhibitors of Membrane Transport and Respiration, When Tested in Phenotype Microarray Profiling

To investigate the impact of PdtaS deficiency on fitness and properties of mycobacterial cells, we applied the BIOLOG Phenotype Microarray screening platform, which allows for convenient testing of growth kinetics of bacteria challenged against multiple antibiotics, detergents, oxidizing agents and toxic compounds. Overall, we exploited 10 PM plates (PM11-20) containing 240 different chemical agents, each arranged in 4 increasing concentrations in a 96-well plate format for simultaneous testing.

When comparing AUCs (the AUC) values of the wild-type *M. smegmatis* and the Δ*pdtaS* mutant, several changes were noted with major alterations in sensitivity profiles to inhibitors of respiration and membrane electron transport, as well as antibiotics targeting the 30S subunit of the ribosome (Supplementary Table S2). The sensitization phenotype of Δ*pdtaS* (AUCs difference > 8500) was observed with the use of aminoglycosides such as tobramycin (>21000), sisomicin, apramycin (>13000), neomycin (>12000), streptomycin (>10000), amikacin (>9000) and gentamicin (>8500). On the other hand, the mutant strain appeared to be more resistant to dihydrostreptomycin (>-22000), another antibiotic targeting the 30S subunit of the ribosome. It is worth noting that amikacin, kanamycin and streptomycin are currently being used as anti-tuberculosis agents, and knowing that their activity may be improved by affecting the expression profile of PdtaS might be useful in future treatment strategies.

The sensitization phenotype of Δ*pdtaS* was also observed with the use of inhibitors of respiration and membrane electron transport. The most potent effect was observed for the inhibitors of respiration such as pentachlorophenol (AUCs difference > 20000), menadione and hexachlorophene (>10000). The potent sensitization phenotype was also observed in the presence of compounds affecting transmembrane proteins (sanguinarine and guanidine hydrochloride, >15000 and 14000, respectively). On the other hand, the Δ*pdtaS* strain was more resistant to other inhibitors of membrane transport and respiration, colistin and iodonitro tetrazolium violet (AUCs difference > -14000).

Additionally, the PdtaS mutant appeared to be more resistant to several additional compounds, including antibiotics such as tetracycline, blasticidin S, penimepicycline, rifampicin, oxacillin, cloxacillin and vancomycin (Supplementary Table S2).

### Inactivation of *pdtaS* Causes Sensitivity of *M. smegmatis* to 30S Targeting Antibiotics

Having identified 30S targeting antibiotics as key agents influencing the survival of *M. smegmatis* lacking *pdtaS* in the Phenotype Microarray, we set out to confirm these observed effects in *M. smegmatis* using spot dilution assays. This relatively simple technique requires spotting 10-fold dilutions of bacteria onto solid growth medium plate containing known amounts of tested antibiotics. Alongside the wild-type and the deletion mutant strain, we included a complementation strain carrying an intact *pdtaS* gene under *P_tet_* promoter on an external plasmid, while the genomic locus remains disrupted. This was to prove that observed effects can indeed be attributed to the inactivation of *pdtaS* as a single causing factor and ectopic expression of PdtaS should reverse the observed phenotypes back to the wild-type patterns. We have chosen to test different ranges of concentrations of apramycin, sisomicin, streptomycin, dihydrostreptomycin and kanamycin, as the mode of action for these antibiotics is well characterized (Supplementary Figure S3A), and they are known to be stable on solid media plates. In fact, kanamycin and streptomycin are used in routine molecular microbiology practice as genetic markers.

The spot dilution assay confirmed the sensitivity patterns previously observed in the Phenotype Microarray for all chosen antibiotics, with the exception of dihydrostreptomycin (Supplementary Figure S3B). Interestingly, in contradiction to the Phenotypic Microarray analyses, *M. smegmatis* Δ*pdtaS* cells showed reduced survival in the presence of dihydrostreptomycin, confirming the sensitization of the Δ*pdtaS* mutant to all tested aminoglycosides.

**FIGURE 1:**
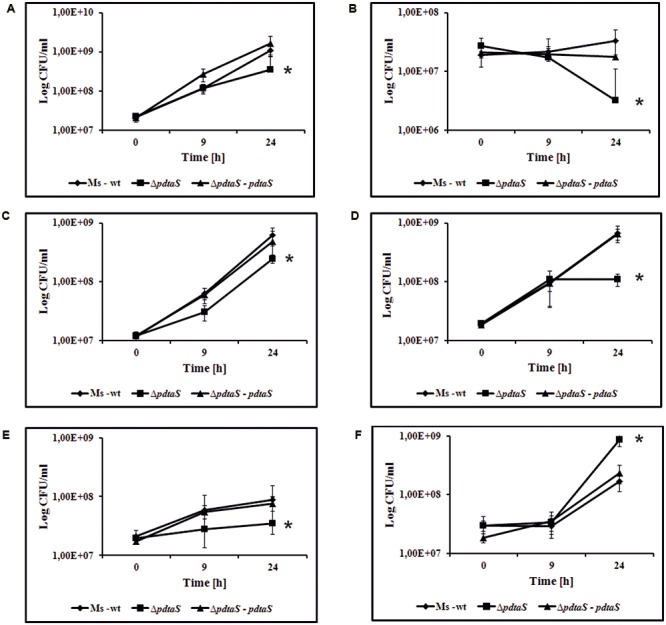
PdtaS deficiency alters sensitivity of *M. smegmatis* cells to various aminoglycoside antibiotics. The *M. smegmatis* wild-type (Ms-wt), mutant Δ*pdtaS* and Δ*pdtaS* complemented strain (Δ*pdtaS*–*pdtaS*) were propagated in 7H9 medium supplemented with OADC. Time-dependent CFU/ml was determined after 9 and 24 h of cell exposure to aminoglycoside antibiotics: **(A)** 0.55 μg/ml apramycin, **(B)** 2.3 μg/ml sisomicin, **(C)** 0.5 μg/ml streptomycin, **(D)** 0.5 μg/ml dihydrostreptomycin, **(E)** 2.0 μg/ml kanamycin, **(F)** 0.1 μg/ml tetracycline. Colony formation values are means ± standard errors from three independent experiments. The statistical analyses were performed using the Student’s *t*-test (apramycin, *P* = 0.012; streptomycin, *P* ≤ 0.001; sisomicin, *P* = 0.007; dihydrostreptomycin, *P* ≤ 0.001; kanamycin, *P* ≤ 0.001; tetracycline, *P* = 0.009).

To further confirm the observed results, the viable colony-forming units per milliliter (CFU/ml) of Δ*pdtaS* mutant cells in the presence of antibiotics were determined. CFU data indicate a significant reduction in viability of Δ*pdtaS* cells compared to the wild-type and the complementation strain in the presence of all aminoglycoside antibiotics tested in liquid medium. After 24 h of growth, the survival of Δ*pdtaS* cells in the presence of kanamycin (2.0 μg/ml) or streptomycin (0.5 μg/ml) was reduced by approximately 60% compared to the wild-type strain. Addition of apramycin (0.55 μg/ml) or dihydrostreptomycin (0.5 μg/ml) resulted in growth inhibition of mutant cells by approximately 75 or 80%, respectively, in respect to the control strain. Sisomicin (2.3 μg/ml) affected the survival of Δ*pdtaS* by approximately 90% in comparison to the wild-type strain. Interestingly, the Δ*pdtaS* mutant strain was more resistant to the presence of tetracycline (0.1 μg/ml) in the growing medium, in comparison to the wild-type strain. The survival of *M. smegmatis* wild-type cells was inhibited by about 65% in comparison to Δ*pdtaS* cells. Concentrations of antibiotics used for CFU assessment were chosen based on results obtained in the spot dilution assay. The obtained data for all antibiotics used in this study, were considered statistically significant using Student *t*-test (**Figure 1**). All the observed phenotypes were fully reversible when a complementing copy of *pdtaS* gene under its native promoter was introduced into the Δ*pdtaS* mutant strain. Finally, the minimal inhibitory concentration (MIC) for the all tested antibiotics was determined using microplate Alamar blue assay (MABA). In this assay, the presence of mycobacterial growth causes the redox indicator Alamar blue to turn from blue to pink (Leonard et al., 2008). The obtained MIC values are presented in **Table 1**. The results confirmed significant sensitization of the PdtaS deficient strain to kanamycin, streptomycin and dihydrostreptomycin. Surprisingly, the MIC values for apramycin and sisomycin remained on the wild-type level, despite the BIOLOG, CFU analysis and spot assay results all indicating growth defect of PdtaS mutant strain, when cultured in the presence of tested antibiotics. Results obtained from MIC for tetracycline confirmed previous observations (BIOLOG, CFU and spot dilution assay) that the Δ*pdtaS* strain is more resistant to tetracycline in comparison to the wild-type strain.

**Table 1:** Minimum inhibitory concentration (MIC) of antibiotics used in that studies.

	MIC (μg/ml)		
	Ms-wt	Δ*pdtaS*	Δ*pdtaS* - *pdtaS*
Kanamycin	1.5	0.75	1.5
Streptomycin	0.625	0.312	0.625
Dihydrostreptomycin	0.312	0.156	0.312
Apramycin	1.25	1.25	1.25
Sisomicin	2	2	2
Tetracycline	0.25	0.5	0.25

The MIC obtained for kanamycin was 1.5 μg/ml for wild-type and complimenting strain and 0.75 μg/ml for the mutant strain. Analysis for other antimicrobial agents showed considerably lower MIC than for kanamycin. MIC for streptomycin was 0.625 μg/ml for Ms-wt and Δ*pdtaS* – *pdtaS* strains and 0.312 μg/ml for Δ*pdtaS* strain. MIC for dihydrostreptomycin is 0.312 μg/ml for Ms-wt and Δ*pdtaS* – *pdtaS* strains and 0.156 μg/ml for Δ*pdtaS* strain. Values of MIC for apramycin and sisomicin are equal to all tested strains and they are respectively 1.25 μg/ml and 2 μg/ml. The obtained MIC for tetracycline was 0.5 μg/ml for the Δ*pdtaS* strain and 0.25 μg/ml for the wild-type and complementing strains.

This clearly indicates that the activity of PdtaS as a single causing factor is indeed responsible for the observed sensitization of the mutant strain to aminoglycoside antibiotics.

### Removal of PdtaS Moderately Influences Oxidative Respiration Efficiency

**FIGURE 2:**
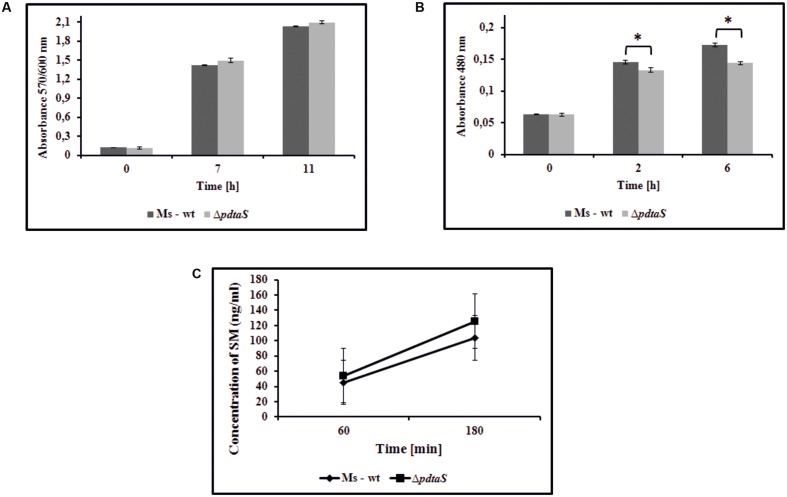
Insufficiency of PdtaS affects the oxidative respiration efficiency. **(A)** Time dependent Alamar blue metabolic activity of the reduced form of resazurin by wild-type and Δ*pdtaS M. smegmatis* cells. Absorbance at 570/600 nm values are means ± standard errors from three independent experiments. **(B)** The *in vivo* reduction of redox indicator – TTC in wild-type and Δ*pdtaS M. smegmatis* strains. Absorbance at 480 nm values are means ± standard error from three independent experiments. All means marked with ^∗^(*p* < 0.001) are significantly different from the control in Student’s *t*-test analysis. **(C)** The uptake of streptomycin by wild-type and Δ*pdtaS* cells monitored by competitive ELISA immunoassay. The bacterial cells were exposed to SM (0.7 μg/ml) for 60 and 180 min and concentration of intracellularly deposited antibiotic was measured. The mean ± standard deviation values from three independent experiments are shown.

The data mining indicated that PdtaS may be linked to reaeration and oxygen sensing. On the other hand, from the Phenotype Microarray platform, we have observed alterations in sensitivity of the Δ*pdtaS* strain to compounds interfering with the respiratory chain. It was previously shown that electron motive force can influence sensitivity or resistance patterns against aminoglycosides (Taber et al., 1987). To verify if altered oxidative respiration efficiency was responsible for the observed antibiotic sensitivity patterns in the Δ*pdtaS* strain, we utilized the Alamar blue assay and 2,3,5-thriphenyl tetrazolium chloride reduction assay (TTC assay), commonly used redox indicators of cellular respiration. *M. smegmatis* Δ*pdtaS* cells did not show any significant changes in the ability to utilize resazurin as the electron acceptor in the electron transport chain compared to wild-type cells (**Figure 2A**). As the reduction of Alamar blue can be connected to various reductive forces in the cell, we have utilized a similar, more targeted method with TTC as the electron acceptor. The reduction of colorless TCC to red TPF (1,3,5-tri phenylformazan) in *M. smegmatis* cells was visible after 2 h of incubation at 37°C. The measurement of absorbance at 480 nm consistently showed decreased utilization of TTC by approximately 9% and 17% of Δ*pdtaS* cells after 2 or 6 h of incubation, respectively, when compared to TTC reduction efficiency measured in wild-type cells (**Figure 2B**).

Among the mechanisms that could contribute to the sensitization of Δ*pdtaS* to aminoglycosides, one should consider the efficiency of uptake of these antibiotics and the intracellular inactivation of these drugs via enzymatic conversion into inactive derivatives or via drug efflux mechanisms (Garneau-Tsodikova and Labby, 2016). Likewise, the uptake of aminoglycosides depends on the efficiency of an electron flow through the respiratory chain, which could be modestly affected in Δ*pdtaS* as indicated by a tetrazolium reduction assay. To assess the above possibilities, we have determined the concentrations of intracellularly deposited streptomycin by the wild-type and Δ*pdtaS M. smegmatis* cells pre-incubated with this anti-tuberculosis drug. The intracellular levels of the tested aminoglycoside were monitored using the competitive ELISA immunoassay. Exponentially grown bacteria were exposed to SM at a concentration of 0.7 μg/ml for 60 and 180 min. The control bacilli were incubated for the same amount of time without the antibiotic. The resultant cell lysates were used to measure the concentration of SM released from the disrupted cells. The concentration of SM in lysates obtained from wild-type and Δ*pdtaS M. smegmatis* exposed to the antibiotic for 60 min enriched 45.35 (±15.83) and 54.22 (±5.18) ng/ml, respectively. However, the intracellular concentrations of SM in the lysates obtained from cells exposed to this drug for additional 2 h (180 min) were significantly higher and were calculated as 103.89 (±19.94) and 125.45 (±11.45) ng/ml for wild-type and mutant lysates, respectively (**Figure 2C**). The concentration of SM in the control mycobacterial cells (growing without an antibiotic) fell below 1 ng/ml at both experimental time points of 60 and 180 min. Based on the above data, we concluded that the uptake of SM was not significantly affected in the Δ*pdtaS* mutant cells, and the sensitization of the mutant to aminoglycosides was not due to an increased concentration of intracellularly deposited antibiotics.

### The rRNA Expression Levels Are Not Affected in the Δ*pdtaS* Mutant

**FIGURE 3:**
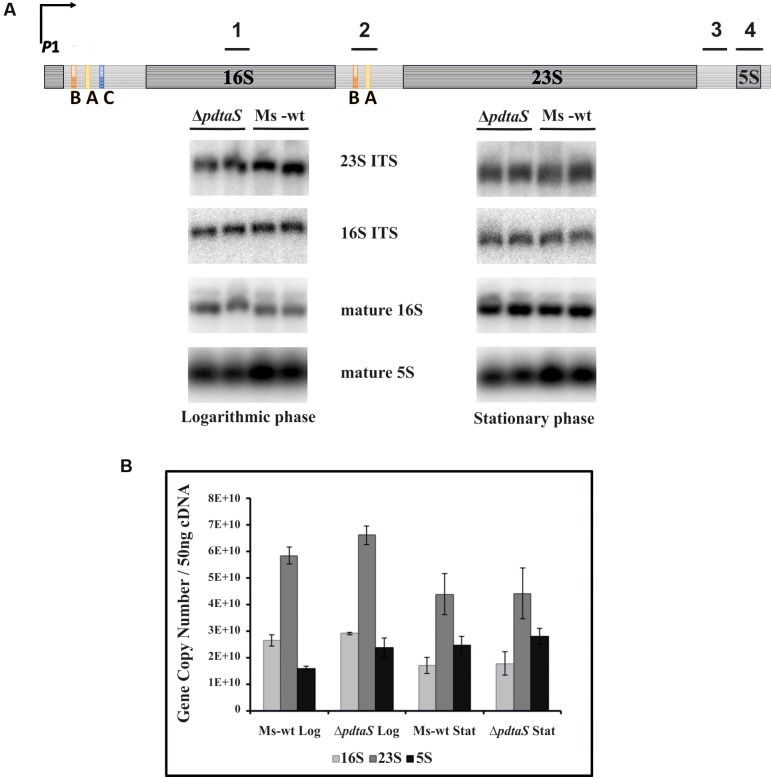
The expression levels of the *rrn* is not significantly affected by deficiency of PdtaS. **(A)** Schematic of antitermination within the *M. smegmatis rrn* operons is shown with marked Northern hybridization sites: 1-16S mature; 2-16S ITS; 3-23S ITS; 4-5S mature. 2 μg of total RNA from wild-type or Δ*pdtaS* cells from logarithmic and stationary phases of growth were resolved on a 2% agarose gel under denaturing conditions. Mature 16S and 5S, as well as precursor p16S and p23S, were monitored by Northern blotting analysis with probes complementary to mature or ITS regions, respectively. **(B)** qRT-PCR analysis of 16S, 23S and 5S transcript levels in *M. smegmatis ΔpdtaS* and wild-type cells. Total RNA was isolated from cells collected during logarithmic and stationary phases of growth, reverse transcribed and subjected to qRT-PCR using SYBR green chemistry. Relative amounts of gene expression were calculated by standard curve method. The values are presented as copy numbers per 50 ng cDNA used. Log refers to cells at logarithmic phase of growth, whereas Stat refers to cells at stationary phase. RNA samples for this study were isolated from three independently grown cultures. Mean values and standard deviations from three independent experiments are shown.

Under optimal growth conditions, i.e., rich laboratory culturing media, rRNA transcription in bacteria proceeds as a single polycistronic transcript without interruptions, resulting in proper secondary structure folding and maturation of the transcribed rRNA into three equally abundant species: 16S, 23S and 5S. The pre-rRNA transcript contains relatively long 5′ UTR and the regions encoding mature fragments are separated by intratranscribed spacer regions (ITS). The 5′ UTR and the ITS1 between 16S and 23S contain defined antitermination boxes, which require binding of antitermination factors in order to prevent RNA polymerase pausing or premature disengagement during *rrn* operon transcription (**Figure 3A**). In other microorganisms, as well as mycobacteria, these boxes are known to bind proteins of the Nus antitermination complex, as well as yet unknown additional antitermination factors (Arnvig et al., 2004; Beuth et al., 2005).

The non-coding regions not only serve as mechanism for antitermination driven regulation of *rrn* expression but also influence the maturation pathway that will lead to production of the final rRNA species. They are partially self-complementary, allowing processing of the resulting double stranded RNA by RNase III to initiate appropriate maturation of rRNA. Thus, lack of antitermination may not only produce shorter pre-rRNA species but may also lead to aberrant or alternative maturation. On the other hand, changes in the rRNA itself could translate into altered sensitivity or resistance to aminoglycosides. Since PdtaR, a cellular partner of PdtaS, is believed to be involved in antitermination, we have decided to assess isolated RNA profiles of *M. smegmatis* and the Δ*pdtaS* mutant. Carrying out Northern blotting with radiolabeled oligonucleotide probes able to hybridize specifically to mature 16S or 5S species or 16S and 23S precursors (complementary to corresponding ITS regions), we controlled for the appropriate rRNA species and their precursors. Under the conditions tested, we failed to observe significantly altered ratios of these non-coding RNA species between the tested strains. We could observe somewhat decreased levels of the mature 5S for the mutant RNA profiles on the Northern blots with 5S binding probes (**Figure 3A**). To cross-validate the above results, we evaluated 16S, 23S and 5S transcription in Δ*pdtaS* and *M. smegmatis* wild-type cells by qRT-PCR relative to specific gene copy numbers. Total RNA was extracted from cells at logarithmic and stationary phases of growth following a previously published protocol (Pawelczyk et al., 2011). The collected data revealed no significant changes in 16S, 23S, and 5S transcript levels for *M. smegmatis* cells obtained from both logarithmic and stationary phases of growth (**Figure 3B**). Thus, we do not consider these results as indicative of dramatic problems with the antitermination of the *rrn* operons in *M. smegmatis* lacking PdtaS.

### Altered Ratio of Ribosomal Subunits and Differences in 30S Protein Content May Be the Main Causes of Unusual Antibiotic Resistance Patterns of the Δ*pdtaS* Mutant

Previous studies on *rrn* antitermination in *E. coli* suggested that the polar expression of precursor rRNA species has a more apparent impact on the ribosomal subunits ratio in the bacterial cell than it has on rRNA levels (Quan et al., 2005). To check whether removal of PdtaS causes any changes to the quantity and quality of the ribosomal subunits, we isolated ribosomes from *M. smegmatis* and the respective Δ*pdtaS* strain and resolved them on linear gradients of sucrose using centrifugal force.

**FIGURE 4:**
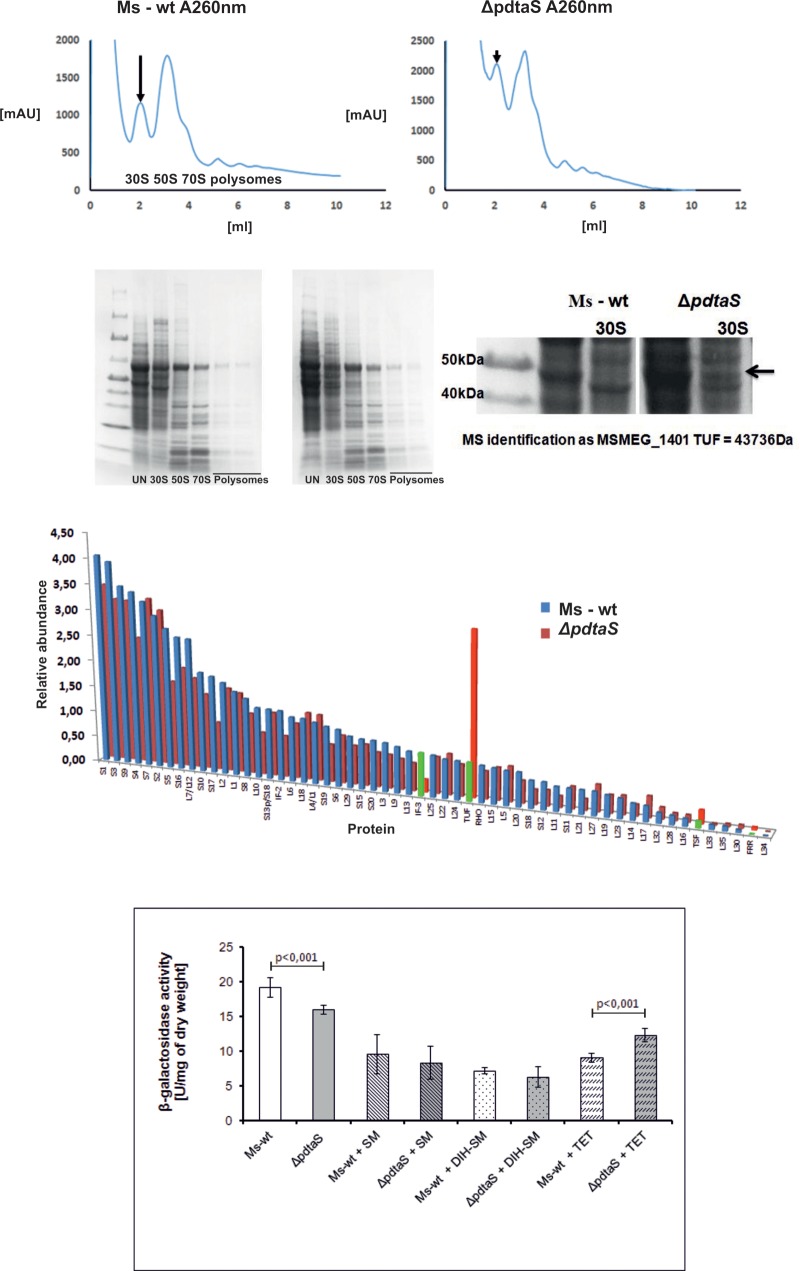
PdtaS deficiency affects 30S:50S ratio and composition of ribosomal subunits in mycobacteria. **(A)** Resulting accumulation of 30S ribosome in the strain lacking PdtaS was assessed by examining intracellular ratios of 30S to 50S ribosomal subunits in lysates from exponentially grown *M. smegmatis* strains resolved by ultracentrifugation of cellular lysates on linear gradients of sucrose. Signal monitored at 260 nm was recorded. Arrows indicate the differences in the levels of 30S subunit between the wild-type and mutant strains. **(B)** SDS-PAGE gel representing fractions containing ribosomal subunits from Δ*pdtaS* and wild-type cell extract. **(C)** Mass spectrometry identification of protein fractionations from the sucrose gradient ribosome fractionation of Δ*pdtaS* and wild-type cells. **(D)** β-galactosidase activity assay for measurement of translation rates in the wild-type and the Δ*pdtaS* strains. The results shown are average from three independent experiments.

Under typical circumstances, the 30S small subunit of the ribosome is present within the cell at roughly the same amounts as the large subunit, the 50S. Together they form the 70S subunit while actively translating a transcript, and if there are multiple ribosomes placed on an intact transcript they may be observed as polysomes on sucrose gradients. The mycobacterial small subunit is typically composed of approximately 20 proteins and 16S rRNA, whereas the large subunit is formed of approximately 29 proteins, the large rRNA fragment -23S and small 5S (Shasmal and Sengupta, 2012). The 50S subunit, even though present at an equivalent molecular ratio, is thus emitting more signal when monitored by UV 260 or 280 nm for nucleic acid and protein signal detection, respectively. While this was indeed the case for our wild-type *M. smegmatis* preparations (**Figure 4A**, left side panel), the ribosome profiles from the Δ*pdtaS* mutant very consistently showed elevated signal for the 30S subunit (**Figure 4A**, right side panel). The overall signal height repeatedly exceeded the signal coming from 50S when gradients were monitored at UV 260 nm, indicating the complex mass of 30S subunits may be higher in those cells than the mass from 50S. The difference in signal strength was also very apparent when looking at UV 280 nm for protein signal (data not shown).

Ribosomal subunits isolated during fractionation of mycobacterial lysates on sucrose gradients were further subjected to comparative proteomic analysis. Isolated fractions corresponding to individual ribosomal subunits were resolved on SDS-PAGE gels, and a single visually appealing difference was spotted between ribosomal profiles of the wild-type and mutant strains. A single protein band found to be overrepresented in the 30S subunits of the Δ*pdtaS* was excised from the SDS-PAGE gel (**Figure 4B**) and identified by mass spectrometry analysis as translation elongation factor TUF (Supplementary Table S3). In parallel, the fractions containing ribosomal subunits were also subjected to total proteomic analysis by mass spectrometry. The identification of individual proteins in each sample were conducted using label free quantification with help of MaxQuant software. We confirmed that individual fractions contained appropriate subunits and comparative analysis revealed that indeed TUF is overrepresented about five-fold in the spectra of 30S subunits of the Δ*pdtaS* mutant. In addition to TUF, we have identified three other proteins in the 30S subunit of the mutant that differed from the wild-type strain by at least two-fold. Among them, another translation elongation factor TSF and ribosome recycling factor FRR were more abundant in the 30S subunits of the mutant and initiation factor IF-3 was over 3-fold depleted from the mutant’s proteomic spectra (**Figure 4C** and Supplementary Table S3).

The observed changes in ribosome composition could potentially lead to an altered rate of translation in the investigated strains. To at least partially address this question, we thus decided to test the translation efficiency in an *in vivo* test. We transformed the wild-type and Δ*pdtaS* strains with a plasmid encoding the gene for β-galactosidase enzyme, which activity can be easily monitored and quantified using a colorimetric assay. Similar strategy is applied to quantify the strength of protein–protein interactions in a bacterial two hybrid system (Karimova et al., 2000). Using similar methodology we were able to determine the amount of β-galactosidase that was expressed in the investigated strains, normalizing the results to the optical density of each bacterial culture. We have noticed a highly reproducible and statistically significant decrease in the production of β-galactosidase per OD_600_ unit of cells, in the mutant strain (15.9 ± 0.64), comparing to the wild-type (19.15 ± 1.37), in the absence of antibiotics (**Figure 4D**). The decrease in the production of β-galactosidase was apparent in both, the wild-type and control strains, when bacteria were grown in the media containing aminoglycosides: streptomycin 0.7 (**Figure 4D**), 0.2 and 0.1 μg/ml (data not shown) and dihydrostreptomycin 0.5 (**Figure 4D**), 0.05 and 0.01 μg/ml (data not shown). On the other hand, the addition of tetracycline significantly inhibited the β-galactosidase production in the wild-type but not in the Δ*pdtaS* strain. The mutant strain produced more enzyme (12.34 ± 0.96) than the wild-type (9.02 ± 0.67), when grown in the presence of tetracycline, while the growth rate was comparable. The above data was collected after relatively short treatment with antibiotics, at times and concentrations that did not cause substantial amount of bacterial killing in a broth dilution assay, even after 9 h incubation, assessed by CFU (**Figure 1** and data not shown).

## Discussion

To investigate the possible pathways that the PdtaS/R system could potentially control in the mycobacterial cell, we have generated a directed and unmarked mutant strain lacking PdtaS in the saprophytic cousin of *M. tuberculosis*, *M. smegmatis*. The *M. tuberculosis* PdtaS (Rv3220c) is known to be a non-essential gene and had previously been disrupted by Parish and collaborators, where the knockout strain of H37Rv with a disrupted *pdtaS* gene was used for animal infection studies and did not affect the time to death for the SCID mouse model of tuberculosis (Parish et al., 2003).

To gain insight into the growth conditions at which PdtaS could operate, we have looked into a large number of transcriptional profiles for *M. tuberculosis* grown under various conditions and in the presence of various potentially mycobactericidal agents (Reddy et al., 2009; Galagan et al., 2010). *PdtaS* transcription drops down during macrophage infection (Fontan et al., 2008) and is significantly depleted during hypoxia and in a NAD^+^ production knockout strain lacking the nadABC operon (Rustad et al., 2008; Vilchèze et al., 2010). On the other hand, *pdtaS* transcript increases over 6-fold within the first 4 h of reaeration (Sherrid et al., 2010).

We have exploited the BIOLOG Phenotype Microarray screening technology to initiate the search for phenotypes associated with removal of PdtaS histidine kinase from the mycobacterial cell. The screening platform, in a convenient 96 well plate format, is compatible with saprophytic *M. smegmatis* strains, which have a much faster generation time than pathogenic *M. tuberculosis*. This was advantageous as the functions of most TCSSs are highly conserved among various species of bacteria and can usually be cross-validated between species. A similar approach had been previously used to characterize a multidrug efflux pump component—MSMEG_2631 (mmp), the removal of which resulted in an increased susceptibility to phleomycin, bleomycin, capreomycin, amikacin, kanamycin, cetylpyridinium chloride, and several sulfa drugs (Khatri et al., 2013). In the case of our *M. smegmatis*Δ*pdtaS* strain, we were unable to detect strong links between the absence of the histidine kinase and basic metabolism. The PdtaS mutant was, however, more sensitive to the wide range of aminoglycosides as well as transport and respiration inhibitors. On the other hand, the Δ*pdtaS* appeared to be more resistant to tetracycline antibiotics such as tetracycline and penimepicycline. The sensitivity of the *pdtaS* defective strain to aminoglycosides and tetracycline was also verified by classical microbiological methods, such as determination of survival by monitoring the CFU for the wild-type and mutant strains after exposure to drugs. Kanamycin, apramycin, sisomicin, streptomycin and dihydrostreptomycin were all found to be more effective and tetracycline less effective against the Δ*pdtaS* mutant then the wild-type. Importantly, complementation of the mutant with a plasmid encoding an intact copy of *pdtaS* reversed the observed phenotypes back to wild-type levels. Moreover, using the MABA assay, the MIC value for Δ*pdtaS* strain was significantly lower in the presence of kanamycin, streptomycin and dihydrostreptomycin and higher in the presence of tetracycline, comparing to control strain. In the case of apramycin and sisomicin we did not observe the difference in MIC value between studied strains. This may be due to the small difference in susceptibility of this strains to the antibiotics used.

The best hits for antibiotics affecting the growth of the Δ*pdtaS* strain were aminoglycoside and tetracycline antibiotics known to interfere with the 30S subunit of the ribosome (Wilson, 2014). In all but the case of dihydrostreptomycin, the results obtained from the BIOLOG screening platform were consistent with the classical bacteriological assessments of bacterial viability. The presence of dihydrostreptomycin on BIOLOG plates was found to favor metabolic activity of the mutant strain over the wild-type *M. smegmatis*, whereas this 30S targeting antibiotic inhibited the growth of the mutant more efficiently than the wild-type when incubated in the growth media before plating CFU. The kinetic plot analysis of control plates (*M. smegmatis* wild-type) PM19 for G06-8 carrying different concentrations of dihydrostreptomycin showed significant differentiations in particular repeats, which might explain the discrepancies between microarray and classical growth analysis in the case of this antibiotic.

In general, the strain lacking PdtaS was more sensitive to aminoglycosides and more resistant to tetracyclines, drugs affecting 30S subunit of ribosomes. Thus, further investigation of the underlying mechanism of sensitization of the Δ*pdtaS* strain to 30S targeting drugs was continued. There are several mechanisms that contribute to the bacterial resistance/sensitivity profiles against aminoglycosides and tetracyclines. Among them are mutations or modifications of rRNA, qualitative and quantitative rearrangements of the ribosomal composition, drug uptake or efflux mechanisms and finally enzymatic inactivation of antibiotics. We attempted to systematically test the most likely hypothesis behind the observed bactericidal effects. We initially explored the potential of rRNA molecules to confer sensitivity against chosen antibiotics. This notion was particularly attractive as it has been proposed that the ribosomal RNA must be undergoing extensive antitermination in order to prevent polar expression of its species and to allow the appropriate RNA structure folding needed for its proper maturation (Gourse et al., 1996; Kaczanowska and Ryden-Aulin, 2007). Studies from *E. coli* showed that most of the *rrn* operons have present antitermination boxA, boxB, and boxC sequences downstream to the P2 transcription start site (promoter 2) and a second set of antitermination boxes within the ITS between the 16S and 23S rRNA sequences, which both are processed via NusA mediated antitermination (Kaczanowska and Ryden-Aulin, 2007). Antitermination within the *rrn* operons is ubiquitous among multiple bacterial species and boxA, B and C sequences of *M. tuberculosis* had been determined to be present within the leader and ITS sequences of the *rrn* molecule in this pathogen (Berg et al., 1989; Beuth et al., 2005). In *E. coli*, as well as in mycobacteria, the *rrn* operons are under the control of the two promoters P1 and P2 or P1 and pCL1, respectively. Most bacterial species encode for multiple *rrn* operons on their genomes, however, *M. tuberculosis* encodes for only a single *rrn* operon. *M. smegmati*s, on the other hand, encodes for two *rrn* operons and can drive expression of each of them from at least 3 distinct promoters (Gonzalez-y-Merchand et al., 1996). We applied Northern blotting and qRT-PCR to detect the levels of expression of precursors and mature species of rRNA in the wild-type and Δ*pdtaS M. smegmatis.* While we could observe a slight difference in the amount of mature 5S between the strains using the Northern blot analysis, we were unable to confirm that polar expression of the *rrn* operon was responsible for this observation judging from qRT-PCR results. We even carried out qRT-PCR reactions with cDNAs generated using random hexamers (data not shown) in parallel with transcript specific primers and still detected no hints of the polar expression of the rRNA species. We concluded that *rrn* antitermination may not be PdtaS dependent and other mechanisms may be responsible for the observed aminoglycoside sensitization phenotype.

The next logical step was to look at the ribosomes themselves. We investigated the ratio of ribosomal subunits 30S/50S in the wild-type and mutant strain, detecting a relative overproduction of the ribosomal 30S subunit in Δ*pdtaS.* The ribosomal subunits were also separated and analyzed by mass spectrometry, revealing that the composition of ribosomal subunits in the mutant strain is significantly affected. Among the most altered ribosome building blocks were the two translation elongation factors TUF and TSF and the ribosome recycling factor RRF, which were all more abundant on the mutant’s 30S subunit. Persistence of elongation factors and ribosome recycling factors on the 30S may suggest that ribosome recycling may be more common in the Δ*pdtaS* than in the wild-type. However, IF-3 was found to be underrepresented on the mutant’s 30S compared to the wild-type strain, and this protein is known to stabilize the transiently recycled 30S subunits by preventing reassociation into 70S in *E. coli* (Hirokawa et al., 2005). Studies in *M. tuberculosis* showed that kanamycin and streptomycin both affect the positioning of the IF-3 on the 30S platform and the overall dynamics of 30S association with the IF-3, thus perturbing the translation initiation (Chulluncuy et al., 2016). Low abundance of IF-3 on the mutant’s ribosomes may indicate that these 30S ribosomal subunits remain in conformation, thus favoring the inhibition of translation by aminoglycosides. Aminoglycosides could possibly further decrease the affinity of IF-3 to the 30S platform and block the translation initiation. Persistence of translation elongation factors TUF and TSF on the ribosomes may suggest that the rate of translation in the Δ*pdtaS* strain is affected as both proteins interact and are needed for efficient guanine nucleotide exchange during peptide chain elongation (Jonak et al., 1998). Indeed, the efficiency of translation measured by β-galactosidase activity was down-regulated in the Δ*pdtaS* strain comparing to the wild-type strain. The addition of aminoglycosides additionally decreased the efficiency of translation in both the wild-type and mutant strain, however, without statistically significant differences between strains. On the other hand, the tetracycline inhibited the β-galactosidase activity normalized to the optical density of cultures more efficiently in a wild-type strain than in the Δ*pdtaS* mutant. Our results indicate that the translation efficiency in the mutant strain is down regulated due to the lack of PdtaS protein, however, the translation process in the mutant strain is less sensitive to the presence of tetracycline, likely because of its lower affinity to the ribosome modified in Δ*pdtaS* strain. We hypothesize that the binding of tetracyclines and/or aminoglycosides to the modified ribosomes is altered in the Δ*pdtaS* mutant, however we are not able to exclude the possibility that the modified protein composition of the mutant’s ribosomes may partially affect their function, making the bacilli more sensitive to some antibiotics targeting 30S subunits.

We have observed resistance of the mutant against tetracycline, penimepicycline and blastocidin S, all binding to or influencing 30S subunit. Ribosomal protection is one of the mechanisms available to confer resistance against tetracyclines, and ribosome protection proteins are homologs of elongation factor TUF, or EF-G, displaying GTPase activity to drive conformational changes of the ribosome and thus make it unavailable for tetracycline binding (Connell et al., 2003). We speculate that persistence of elongation factors TUF and TSF on ribosomes of Δ*pdtaS* could also provide some level of ribosome protection against tetracyclines, keeping the ribosomes in a confirmation that provides resistance to tetracyclines but makes them more available for binding of aminoglycosides.

On the BIOLOG screening platform, Δ*pdtaS* was sensitized not only to aminoglycosides but also inhibitors of respiration and electron transport. This would be consistent with the transcriptomic profiles available for PdtaS expression from *M. tuberculosis* mentioned earlier and would suggest the link between the expression of this protein and availability of oxygen. On the other hand, the uptake of aminoglycosides into bacterial cells requires proton motive force (PMF) that is generated by electron flow through the respiratory chain (Damper and Epstein, 1981). PMF is largely generated by the respiratory complex I, NADH (reduced form of nicotinamide adenine dinucleotide) dehydrogenase (Nuo) and, to some extent, by complex II, succinate dehydrogenase (Sdh) (Simon et al., 2008). It was recently shown that the Fe-S cluster biogenesis machineries play a key role in aminoglycoside resistance by affecting their PMF-energized uptake. As a consequence, aminoglycosides, whose uptake is strongly PMF-dependent, cannot reach the ribosome, their cytoplasmic target (Ezraty et al., 2013). On the other hand, the removal of PdtaS could cause secondary effects, like increased aminoglycoside uptake as the result of affecting an electron flow through the respiratory chain. Activity of aminoglycoside specific efflux pumps or enzymatic inactivation of the drugs could also affect the antibiotic resistance profile. We hypothesized whether PdtaS could influence the cellular levels of PMF and thus interfere with the transport of aminoglycosides through the cell membrane. Oxidative respiration efficiency was determined by Alamar blue and TTC assays, and the streptomycin uptake was monitored to identify the role of aminoglycoside transport in the observed phenotype. We did not observe any significant changes in reduction of resazurin by *M. smegmatis* mutant and wild-type cells in the Alamar blue assay. The TTC assay revealed a rather modest but statistically significant decrease in the rate of reduction of tetrazolium in Δ*pdtaS* compared to the wild-type strain. Metabolic activity of Δ*pdtaS* was also reduced in the presence of iodonitro tetrazolium violet salts on the BIOLOG screen, further confirming that PdtaS activity has an influence on the respiratory chain efficiency. Logically, this should translate into a decreased PMF and decreased uptake of aminoglycosides. However, monitoring the uptake of streptomycin using a competitive ELISA immunoassay did not show significant differences in the levels of intracellularly deposited streptomycin between the mutant and wild-type strains.

Together, we conclude that ribosomal composition and/or the conformational changes associated with it, rather than other effects, are the key features responsible for the change of antibiotic resistance patterns of the Δ*pdtaS*, as the group of compounds targeting ribosome assembly was diverse and oriented toward various steps of the translation process. We believe that the mechanism presented here may be a contributing factor to mycobacterial antibiotic susceptibility during treatment. This is particularly important as streptomycin is currently still considered a first-line drug, and kanamycin and amikacin are the second line of defense used to treat tuberculosis in patients. A potential treatment strategy can be designed using models like the PdtaS knockout system to take advantage of their drug susceptibility and avoid natural drug resistance mechanisms.

## Author Contributions

RP, JD, and AR-G conceived and designed the experiments. KD, RP, AR-G, AŻ, BD, AZ, and PP performed the experiments. KD, RP, AR-G, AŻ, BD, AZ, PP, and JD analyzed the data. KD, RP, PP, and JD wrote the paper.

## Conflict of Interest Statement

The authors declare that the research was conducted in the absence of any commercial or financial relationships that could be construed as a potential conflict of interest.
