# Supplementary material for: PdtaS Deficiency Affects Resistance of Mycobacteria to Ribosome Targeting Antibiotics
Source: Front Microbiol. 2017 Nov 3;8:2145. doi: 10.3389/fmicb.2017.02145 (PMC5676007; doi:10.3389/fmicb.2017.02145)
Supplement: Supplementary file 1 [file Table_1.pdf]

Table S1. List of strains, plasmids and primers used in this study.

**A. Primers used in this study**		
Name of primer	Sequence (5'>3')	Application
**Primers used to amplify DNA for targeted gene replacement**		
Msmeg1918GR1ScaI-F	CAGTACTGATCACCGACTGGCAGCTGGC	*pdtaS* knock-out
Msmeg1918GR2HindIII-R	CAAGCTTCGACCACCGCGTGCAGATG	
Msmeg1918GR3HindIII-F	CAAGCTTGCCAACCACATCCGCGACTC	
Msmeg1918GR4KpnI-R	CGGTACCGCGCGCCCGATTAGGAGTT	
MspdtaS-probe-F	CCTGCAGACCGTCGCCGC	Southern blotting - hybridization probe
MspdtaS-probe-R	CGGCCGTCACGAGCGTCC	
**Primers used for clonings**		
MsmPdtaSKW08_F	CTACTCTCATCGTGGAATCCTGACAGGATCCAGAGGAG CCTGAGTTCGACCCCTCGGTGATCTGCT	*pdtaS* complementation
MsmPdtaSKW08_R	TCTAGGGTCCCAATTAATTAGCTAAAGCTTCTACTGCGC GCCCCGGCTG	
16S_F_pJET_KpnI	TAGGTACCCTCTGACCTGGGGATTTG	Construction of vectors used for normalization of qRT-PCR data
16S_R_pJET_XbaI	TATCTAGAGTTTCCCCATTCGGACATCC	
23S_5S_F_pJET	GTAAGTTCCGACCTGCACGA	
23S_5S_R_pJET	GGGTGATTCCTAATTTGTGTTCG	
**Primers used for Northern blot**		
23S ITS	CCGTGGAGTGTGGTTGCGAG	oligonucleotide probes complementary to *rrn* operons elements

mature 16S	CCACCGGCTTCGGGTGTTACC	
16S ITS	AGCCGGCGCCACTACAGCGCT	
mature 5S	GCGGTGTCCTACTTTTCCACC	
**Primers used for reverse transcription**		
16SRT_RC	CAGGAATTCCAGTCTCCCCT	cDNA synthesis
23SRT_RC	CACCAGGCACTGTCCCTGAA	
5SRT_RC	TTCGGCGGTGTCCTACTTTT	
**Primers used for qRT-PCR**		
16S_F_SYBR	CCGGAATTACTGGGCGTAAA	*16S* rRNA transcripts
16S_R_SYBR	AGTACTCTAGTCTGCCCGTATC	
23S_F_SYBR	CGATACGGTTTGTGTAGGATAGG	*23S* rRNA transcripts
23S_R_SYBR	GGATATACGGTCCGAGGTTAGA	
5S_F_SYBR	GTTACGGCGGTCCATAGC	*5S* rRNA transcripts
5S_R_SYBR	ACCCGGAAGGGTAGTATCAT	
**B. Plasmids constructed for this study**		
Name	Description	Reference
**STRAINS**		
Top10F’	*Escherichia coli* strains	Laboratory stock
Mc^{2}155	*M. smegmatis* wild type	Laboratory stock
M. smegmatis Δ*pdtaS*	*M. smegmatis pdtaS* KO strain	This study
M. smegmatis Δ*pdta*::P_{tet}*pdtaS*	*M. smegmatis pdtaS* KO strain carrying complementation plasmid	This study
**CLONING VECTORS**		

pJET 1.2/blunt	Blunt cloning vector, Amp^R	Thermo Scientific
pMV306H	Mycobacterial integrating vector, Hyg^R	Med-Immune Inc
pMV306K	Mycobacterial integrating vector, Kan^R	Med-Immune Inc
p2NIL	Recombination vector, nonreplicating in mycobacteria, Kan^R	Parish & Stoker, 2000
pGoal17	Source of PacI cassette, Amp^R	Parish & Stoker, 2000
pKW08Lx	Mycobacterial replication vector carrying inducible P_{tet} promoter, Hyg^R	Laboratory stock (Williams et al.,2010)
pKW08Lx-Int	Mycobacterial integrating vector carrying inducible P_{tet} promoter, Hyg^R	Laboratory stock (Williams et al.,2010)
pKD3	1215 bp upstream fragment of *pdtaS* gene cloned in p2Nil vector, Kan^R	This study
pKD4	Δ *pdtaS* and its flanking regions cloned in p2Nil vector, Kan^R	This study
pKD5	pKD4 carrying PacI cassette, Kan^R	This study
pKL5	*pdtaS* gene cloned into pKW08 replicative vector carrying P_{tet} promoter, Hyg^R	This study
pKD10	*pdtaS* under P_{tet} promoter cloned into pKW08 integrative vector, Hyg^R	This study
pKD11	*16S* rRNA gene cloned into pJET 1.2/blunt vector, Amp^R	This study
pKD12	*23S-5S* rRNA gene cloned into pJET 1.2/blunt vector, AmpR	This study
pAB303	*lacZ* gene cloned into pMV306 vector, Kan^R	This study
