# Supplementary material for: PdtaS Deficiency Affects Resistance of Mycobacteria to Ribosome Targeting Antibiotics
Source: Front Microbiol. 2017 Nov 3;8:2145. doi: 10.3389/fmicb.2017.02145 (PMC5676007; doi:10.3389/fmicb.2017.02145)
Supplement: Supplementary file 4 [file Data_Sheet_1.pdf]

# Supplementary materials

## PdtaS deficiency affects resistance of mycobacteria to ribosome targeting antibiotics

Karolina Dadura[1]§, Renata Płocińska[1]§, Anna Rumijowska-Galewicz[1], Przemysław Płociński[1], Anna Żaczek[2], Bożena Dziadek[3], Andrzej Zaborowski[1] and Jarosław Dziadek[1*]

[1] Institute for Medical Biology, Polish Academy of Sciences, Łódź, Poland

[2] Department of Biochemistry and Cell Biology, University of Rzeszów, Rzeszów, Poland

[3]Department of Immunoparasitology, University of Łódź, Łódź , Poland

§ authors contributed equally to this work

***Corresponding author**: Prof. Jarosław Dziadek email: jdziadek@cbm.pan.pl

**Table of contents:**

1. **Table S1: List of strains, plasmids and primers used in this study.**
2. **Table S2: Phenotype Microarray results PM11-20**
3. **Table S3: Mass Spectrometry analysis of ribosomal fractions**
4. **Figures S1, S2, S3 with respective legends**
5. **References**

**Table S1: List of strains, plasmids and primers used in this study.**

**Attached as a separate appendix in PDF format**

**Table S2. Phenotype Microarray results PM11-20**

**Attached as a separate appendix in an excel format**

Numerical AUC values from at least three independent replicates are provided for each condition tested.

**Table S3. Mass spectrometry analyses of 30S ribosome fraction**

**Attached as a separate appendix in an excel format**

Output file generated by MaxQuant software is provided on the Raw data tab. Intensity values provided for each protein identified by MS (at least two peptides) are semiquantitative values from the label-free analysis. Lines of results for ribosomal proteins are marked with yellow (small subunit) or green coloring (large subunit). The recalculated results are shown as a % of total contribution to the sample protein content and were calculated by dividing individual intensity values for each identified protein divided by the total intensity of the entire sample multiplied by 100. Normalized results were used to calculate the ratio of individual proteins between the wild type and mutant strains.

**Figure S1.**

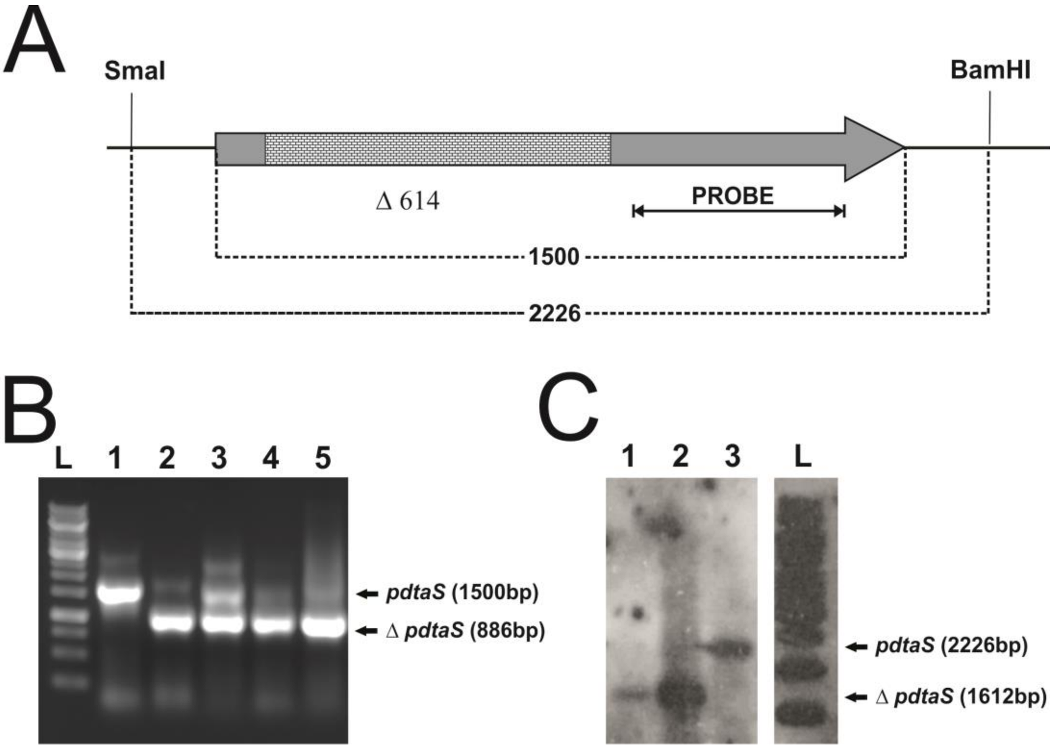


**Figure S1. *M. smegmatis* mutant Δ*pdtaS* preparation strategies. (A)** Schematic showing the restriction-digested DNA fragment (2226 bp) and the size of the internal deletion in the mutated gene (614 bp). The *pdtaS* gene is represented by grey arrows and the internal deletion by lighter rectangles. The *pdtaS* is not essential for the viability of *M. smegmatis*. **(B )** Shown is a PCR confirming the deletion of *pdtaS*. Genomic DNA derived from wild type (WT) strain, single crossover (SCO), double crossover (DCO) and pKD4 plasmid DNA were amplified using *pdtaS* gene-specyfic primers. L- DNA Ladder, 1-WT, 2-pKD4, 3-SCO, 4-DCO1, 5-DCO2. The ladder bands from top to bottom are: 10000 bp, 8000 bp, **6000** bp, 5000 bp, 4000 bp, 3500 bp, **3000** bp, 2500 bp, 2000 bp, 1500bp, **1000** bp, 750bp, 500bp, 250 bp. **(C)** Shown is a Southern blot confirming the deletion of *pdtaS*. Genomic DNA was isolated from WT *M. smegmatis* and two DCO strains, digested with SmaI and BamHI enzymes, transferred to nitrocellulose membrane, and probed with chemiluminescence labeled *pdtaS* fragment. Using this probe, we detected bands corresponding to 1612 bp for mutant and 2226

bp for wild type. Positions of DCO and wild type copy of *pdtaS* bands are marked. 1-DCO, 2- DCO2, 3-WT, L-DNA Ladder.

**Figure S2.**

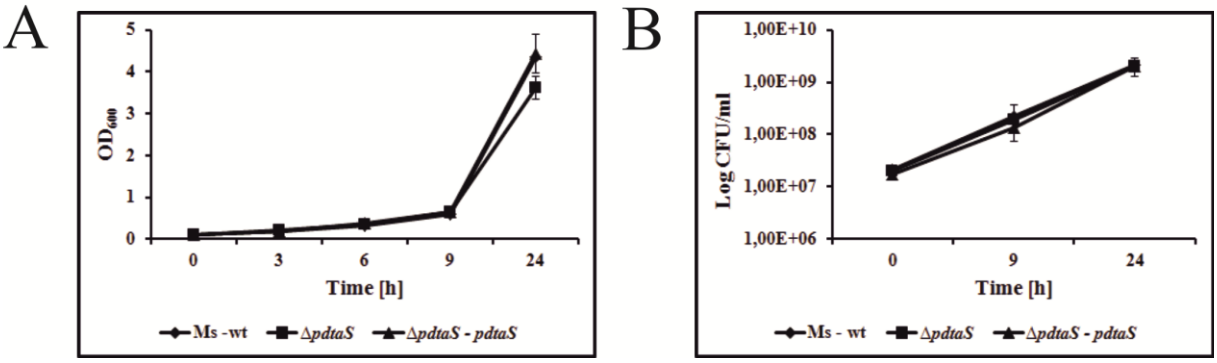


**Figure S2**. **Phenotypic analysis of *M. smegmatis* strains lacking PdtaS sensory kinase. (A)** Growth kinetics analysis of wild-type *M. smegmatis*, Δ*pdtaS* and Δ*pdtaS* strain complemented with an intact copy of *pdtaS* under the control of a tetracycline promoter (Δ*pdtaS–pdtaS)*. Growth rate analyses were performed in rich medium (7H9/ OADC). $OD_{600}$ values are means ± standard errors from three independent experiments. **(B)** Time-dependent colony forming units (CFU/ml) by wild-type *M. smegmatis*, Δ*pdtaS* and Δ*pdtaS-pdtaS* strain. The 10-fold serial dilutions of the cells were prepared and viability was performed in solid medium 7H10 supplemented with OADC. Colony formation values are means ± standard errors from three independent experiments.

**Figure S3.**

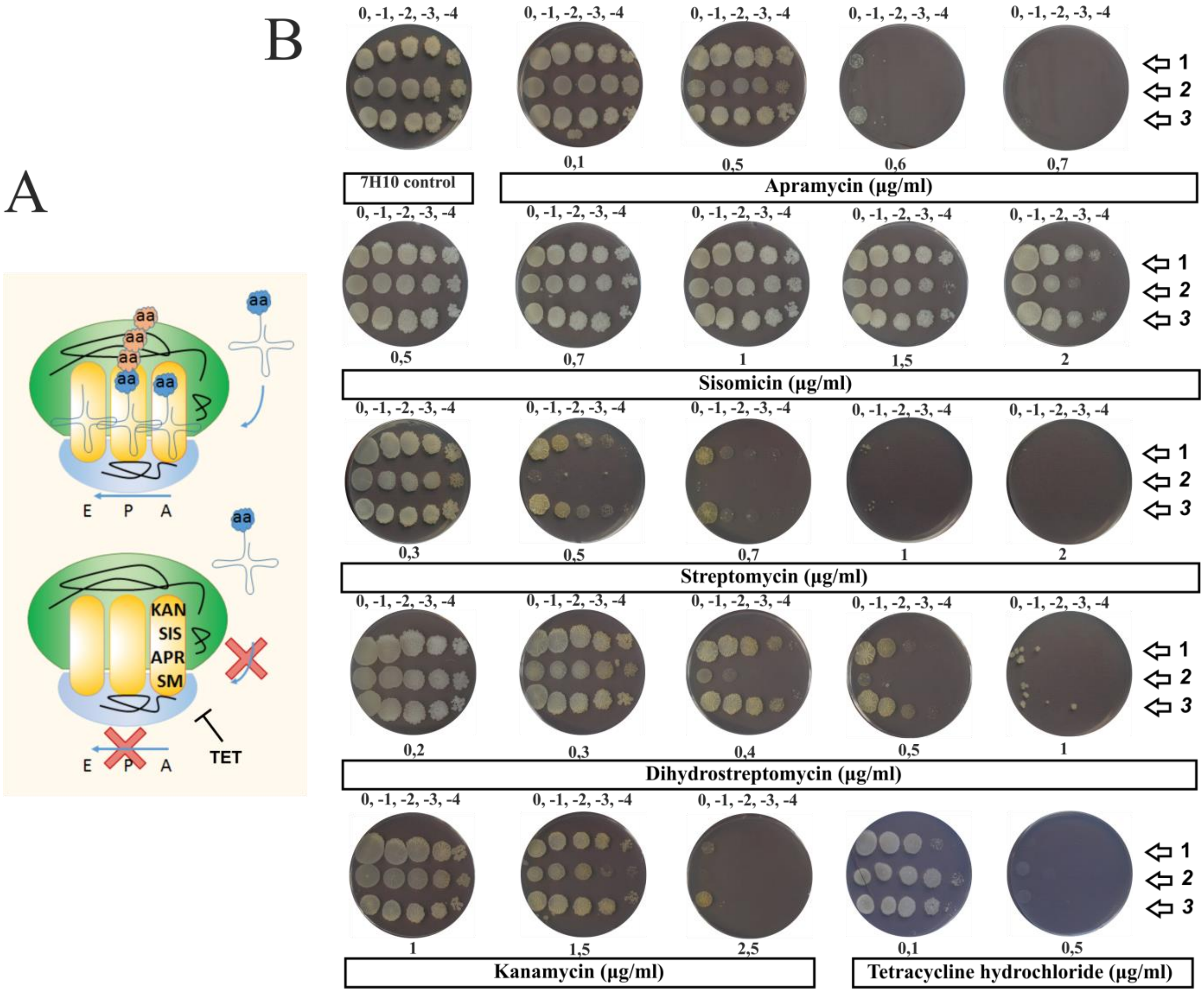


**Figure S3. Deficiency of PdtaS affects levels of intrinsic sensitivity against ribosome targeting antibiotics.** Antibiotics known to interfere with the small ribosome subunit **(A)** were tested in spot dilution assays to assess levels of antibiotic sensitivity of the wild-type (1), Δ*pdtaS* mutant (2) and Δ*pdtaS-pdtaS* strain complemented with an intact copy of *pdtaS* under the control of a tetracycline promoter (3) in *M. smegmatis* **(B).** The presented photos are examples of three biologically independent experiments.

## References

1. **Williams KJ, Joyce G, Robertson BD.** (2010). Improved mycobacterial tetracycline inducible vectors. *Plasmid* 64:69-73.
